# A novel oligo-pyrazole-based thin film: synthesis, characterization, optical and morphological properties

**DOI:** 10.1007/s00396-018-4342-7

**Published:** 2018-06-05

**Authors:** Adnan Cetin, Adem Korkmaz, Ishak Bildirici

**Affiliations:** 1 Faculty of Education, Department of Science, Muş Alparslan University, Muş, Turkey; 2 School of Health, Muş Alparslan University, Muş, Turkey; 3 Faculty of Pharmacy Department of Pharmaceutical Chemistry, Yüzüncü Yıl University, Van, Turkey

**Keywords:** Absorbance, Conjugated polymer, Organic semiconductor, Pyrazole, Thin film

## Abstract

Pyrazole-3,4-dicarboxylic acid **2** was synthesized via the hydrolysis of pyrazole-3-carboxylic acid **1** and subsequently heated with thionyl chloride to give the novel pyrazole-3,4-dicarbonyl dichloride **3**, which was easily converted into oligo-pyrazole **4** upon its reaction with *p*-phenylene-diamine. These newly synthesized compounds were characterized by ^1^H-NMR, ^13^C-NMR, and FT-IR spectroscopy, and gel permission chromatography (GPC). Three novel oligo-pyrazole thin films were prepared using oligo-pyrazole **4** with these respective values of thickness: 20, 21, and 24 μm. The optical properties of the films, including the absorbance, transmittance, and optical band gap, were determined using UV-vis spectroscopy. The *E*_*g*_ values of the films were found to be 1.426, 1.537, and 1.648 eV for the 20, 21, and 24 μm thick organic films, respectively. Atomic force microscopy (AFM) was used to examine the surface morphology and properties of the organic films. In the AFM images, a few black regions were observed and several yellow regions appeared over a large area, and the surface of the oligo-pyrazole films had an extremely low roughness value. The as-synthesized oligo-pyrazole has great potential in optoelectronic applications according to the optical properties of the as-prepared films.

**Electronic supplementary material:**

The online version of this article (10.1007/s00396-018-4342-7) contains supplementary material, which is available to authorized users.

**Figure:**
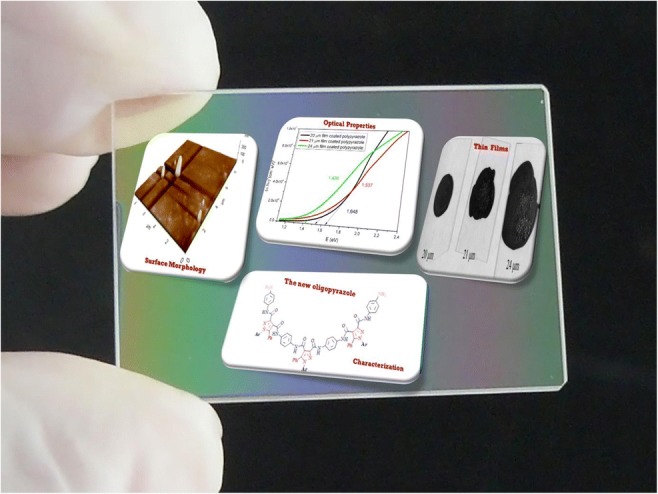
Graphical abstract

## Introduction

Conjugated polymers are important target molecules for synthetic chemists due to their use in solar cells, organic photovoltaics, organic light emitting diodes, and thin film transistors, as well as their industrial applications [1–3]. The search for new materials depends on the rapid development of science and technology, which has created an intense area of research on conducting polymers and increased the importance of the projects in this area. Recently, the synthesis of new polyamide derivatives has become an increasingly popular topic and widely studied research area [4–6]. In particular, their optical properties and physicochemical properties have guided researchers towards the synthesis of polyamide derivatives using existing methods and applications [7, 8].

Poly-pyrazoles are an important class of heterocyclic polymers, which have become more popular than before due to their wide range of properties such as film formation and high thermal stability [9, 10]. Poly-pyrazoles and pyrazoles have also been successfully applied in many fields including biology, industry, and pharmaceutical chemistry [11–13]. Past studies used conjugated polyamides (e.g., amide groups) for drug delivery in medicine [14, 15]. Furthermore, poly-pyrazoles and pyrazoles have many advantages in several application fields such as optics and optoelectronic technology [9, 16].

One of the most common applications of polymer materials is in thin film devices. Thin films that are made of these polymers have several advantages when used as optical coatings, coatings in electronics, and decorative protective coatings, thanks to the fundamental characteristics they have. For instance, optical, mechanical, and electrical properties are significantly increased when polymer materials are used as a thin film [17, 18]. In particular, thin films have been used in the construction of semiconductors and superconducting devices, insulation and transmission coatings, and circuits due to their electrical properties. They are also used in reflective and non-reflective coatings, interference filters, and optical discs due to their optical properties. In addition, thin films are used in memory disks due to their magnetic properties [19, 20].

This study aimed to synthesize different oligo-pyrazole-based thin films that are newly developed and to investigate their optical properties such as the optical band gap and transmittance. Furthermore, the study determined the surface morphology of the new thin film (21 μm) that had different thickness levels using atomic force microscopy (AFM).

## Experimental

### Materials and equipment

All reagents and solvents were purchased from Merck, Sigma and Aldrich companies. These materials were used without purification. Infrared spectra were recorded on a Shimadzu IR-470 spectrophotometer. ^1^H (400 MHz) and ^13^C (100 MHz) NMR spectra were recorded on a Bruker DRX-400 high-performance digital FT-NMR spectrometer. NMR spectra were obtained in solutions of deuterated chloroform. Molecular weight and PDI of the synthesized oligo-pyrazole were determined by gel permeation chromatography (GPC) using Agilent 1100 Series, equipped with refractive index detector. Optical measurements of thin films at different thicknesses were carried out with a Shimadzu model UV-1800 spectrophotometer in the wavelength range of 1100–190 nm at room temperature. An Ambios Q-Scope AFM device was used to study the surface structures of the films.

### 1-(3,4-Dimethylphenyl)-4-(ethoxycarbonyl)-5-phenyl-1*H*-pyrazole-3-carboxylic acid (1)

To synthesize compound **1**, procedure existed in literature was followed [21]. The yield of **1** was 75%; mp 163.02 °C.

### 1-(3,4-Dimethylphenyl)-5-phenyl-1*H*-pyrazole-3,4-dicarboxylic acid (2)

Compound **1** (0.364 g, 1 mmol) was heated in solution of sodium hydroxide (0.1 g 2.5 mmol) in 20 mL water for 1 h. The solution was cooled down to room temperature. It was added with concentrated hydrochloric acid (1.5 mL) and water (1.5 mL). The white solid product was occurred. It was filtered and it was washed with water. Yield 90%. Color white. mp 126–128 °C. FT-IR (ν, cm^−1^) 3350 (br, -OH), 3064 (aromatic C-H), 2936 (aliph. C-H), 1721–1710 (C=O, acide), ^1^H NMR (400 MHz, CDCl_3_) δ (ppm) 11.6 (*br.s*, 2H, -OH), 8.1 (m, 1H), 7.9 (m, 2H), 7.6 (m, 2H), 7.1 (m, 3H), 2.1 (s, 3H, Ar-CH_3_), 1.8 (s, 3H, Ar-CH_3_). ^13^C NMR (100 MHz, CDCl_3_) δ (ppm) 171.3, 167.9 (C=O, acid), 145.2 (C_3_), 142.1 (C_5_), 135.0, 134.0, 132.9, 132.8, 132.0, 128.0, 127.2, 125.0, 115.8, 115.7 (C_4_), 29.4 (Ar-CH_3_), 20.7 (Ar-CH_3_). (+)ESI-HRMS *m*/*z* calculated for [C_19_H_16_N_2_O_4_ + H^+^] 337.3562; observed 337.3565.

### 1-(3,4-Dimethylphenyl)-5-phenyl-1*H*-pyrazole-3,4-dicarbonyl dichloride (3)

Compound 2 (0.397 g, 1 mmol) was added to the reaction flask that included excessive thionyl chloride and refluxed at 80 °C. After 6 h, the reaction mixture, which dissolved over time, was cooled down to room temperature. Excessive thionyl chloride was evaporated. The remaining oily product was purified in dry ether and then was crystallized from toluene, to yield 75%. Color milk white. FT-IR (ν, cm^−1^) 3062 (aromatic C-H), 2928 (aliph. C-H), 1720, 1700 (C=O, acyl), ^1^H NMR (400 MHz, CDCl_3_) δ (ppm) 8.0 (s, 1H), 7.8 (s, 1H), 7.6 (m, 2H), 7.4 (m, 2H), 7.2 (m, 2H), 2.6 (s, 3H, Ar-CH_3_), 2.1 (s, 3H, Ar-CH_3_). ^13^C NMR (100 MHz, CDCl_3_) δ (ppm) 171.0, 167.5 (C=O, acyl), 142.3 (C_3_), 141.8 (C_5_), 135.6, 133.6, 133.0, 132.4, 130.5, 130.1, 128.4, 128.1, 127.7, 126.3, 114.1 (C_4_), 30.8(Ar-CH_3_), 22.8 (Ar-CH_3_). (+)ESI-HRMS *m*/*z* calculated for [C_19_H_14_Cl_2_N_2_O_2_ + H^+^] 374.2438, observed 374.2439.

### Synthesis of poly(p-phenylene-1-(3,4-dimethylphenyl)-5-phenyl-1*H*-pyrazole-3,4-dicarboxyamide (4)

Pyrazole-3,4-dicarbonyl dichloride **3** (0.08 g 0.25 mmol) was dissolved in anhydrous tetrahydrofuran (10 ml). The *p*-phenylene-diamine (0.028 g 0.25 mmol) was added to the reaction pot and the mixture was refluxed for 24 h under an atmosphere of nitrogen. Then, it was cooled to room temperature. The solvent was evaporated. The precipitated product was washed with diethyl ether. Then, it was filtered and dried. Yield 80%. Color brown. FT-IR (ν, cm^−1^) 3385 (N-H, amide), 2967 (aromatic -CH), 1731 (C=O, acyl), 1652 (C=O, amide), 1595–1579 (C=N), 1536–1449 (aromatic, C=C), 1313 (C-N). ^1^H NMR (400 MHz, CDCl_3_) δ (ppm) 8.7, 8.4 (-NH, amide), 7.9–6.3 (m, Ar-H), 4.4 (-NH, aromatic), 2.5, 2.2, 2.1, 2.0, 1.7 (s, Ar-CH_3_). ^13^C NMR (400 MHz, CDCl_3_) δ (ppm) 172.5, 165.8, 162.3, 159.9 (-C=O), 149.8 (C_3_), 142.1 (C_5_), 140.6, 139.6, 139.3, 139.2, 134.5, 130.4, 130.2, 130.1, 129.4, 127.2, 123.1, 121.7, 117.5, 114.1, 111.3, 110.3, 108.3 (C_4’_), 30.9, 30.3, 22.4, 22.7, 21.5 (Ar-CH_3_). *M*n = 1333 g/mol, Mw = 2347 g/mol, (polydispersity index, PDI, 1.872).

### Preparation of the thin films that synthesized oligo-pyrazole (4)

**Fig. 1:**
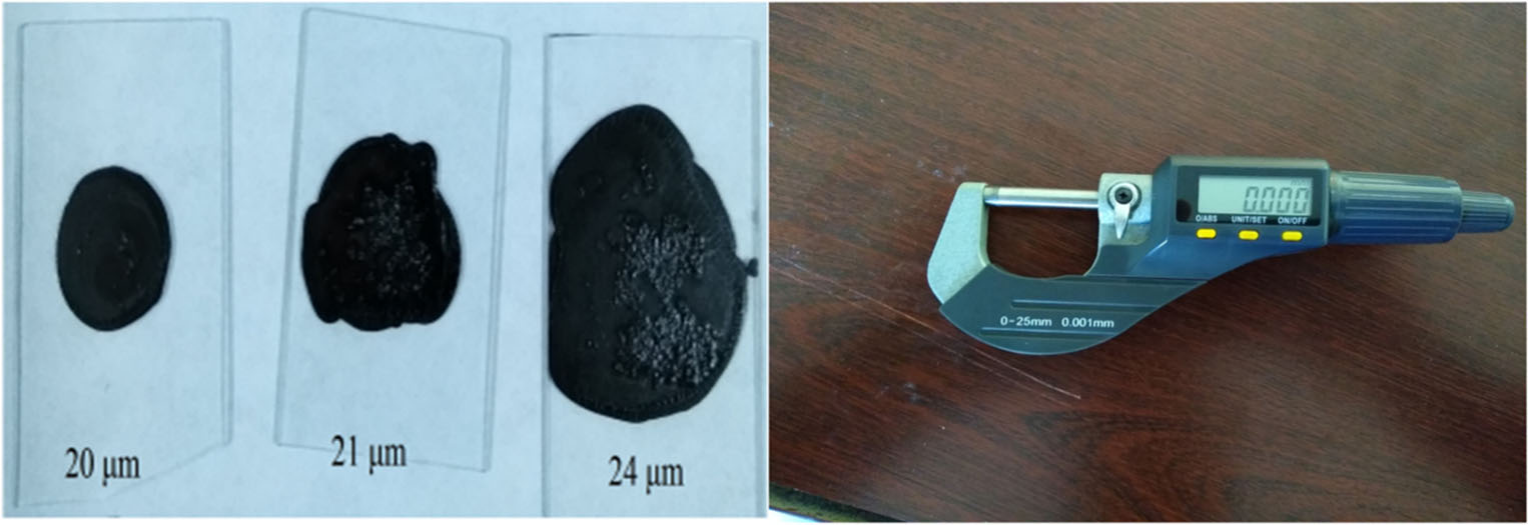
Images of the thin films of oligo-pyrazole with thickness values of 20, 21, and 24 μm

Oligo-pyrazole **4** (0.05 g) was added to 1 mL of DMF and the resulting solution was stirred at room temperature for 1 h. Any insoluble oligo-pyrazole was removed by filtration. The surface of a glass substrate was cleaned using piranha solution (sulfuric acid and hydrogen peroxide) and then rinsed with water. The solution of oligo-pyrazole was added dropwise on the glass substrate and left to dry to form a thin film of oligo-pyrazole **4**. To obtain films with different thickness levels, the process was repeated several times for each film. Using this method, three films with thickness values of 20, 21, and 24 μm were obtained. The thickness values of the films were measured using a micrometer (sensitivity = 0.001 mm), as shown in Fig. 1.

## Results and discussion

### Synthesis and characterization

**Scheme 1:**
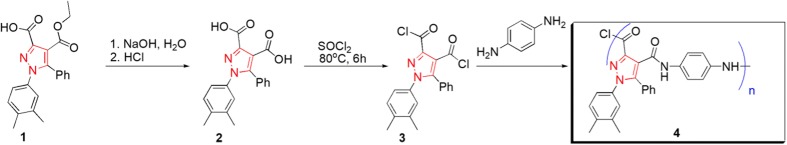
The synthesis of oligo-pyrazole **4**

Poly-pyrazoles are the most extensively studied subject in polymer chemistry and organic chemistry because of their reliability, accessibility, and chemo-selectivity. Pyrazole-3-carboxylic acid **1** was synthesized according to a literature procedure [21] and pyrazole-3,4-dicarboxylic acid **2** was obtained from the basic hydrolysis of **1** (Scheme 1). The structure of **2** was confirmed using NMR spectroscopy by the two carboxylic acid proton signals at *δ* = 11.68 ppm and the two carbonyl carbon signals at *δ* = 171.3 and *δ* 167.9 ppm, and using FT-IR spectroscopy by the characteristic IR absorption bands at 3350 cm^−1^ (COOH), 3064 cm^−1^ (Ar-C-H), 1721 and 1710 cm^−1^ (acid, C=O).

The monomeric starting material, pyrazole-3,4-dicarbonyl dichloride **3**, was prepared upon heating **2** with excess thionyl chloride. All the new compounds (**1–3**) were confirmed using spectroscopic methods, which were consistent with the previous studies [22]. The preparation of oligo-pyrazole was performed using the one-step procedure shown in Scheme 1. Poly(p-phenylene-1-(3,4-dimethylphenyl)-5-phenyl-1H-pyrazole-3,4-dicarboxyamide **4** was synthesized upon the reaction of **3** and *p*-phenylene-diamine in refluxing THF for 1 day under an atmosphere of argon gas. The average molecular weight (*Mn*), average molecular weight (*Mw*), and polydispersity index (PDI) of the as-synthesized oligo-pyrazole **4** were determined by GPC using poly(methyl methacrylate). The Mn, Mw, and PDI of the as-synthesized oligo-pyrazole were observed to be 1333, 2347, and 1.872 g mol^−1^, respectively. As shown Scheme 1, the as-synthesized oligo-pyrazole **4** was determined be a trimeric structure using gel permeation chromatography. The as-synthesized oligo-pyrazole **4** has three pyrazole structural units and ten phenyl groups. In addition, the as-obtained oligo-pyrazole was asymmetric due to the structure of the monomer. The better conjugation was used as a warranty since there were many aromatic structures. This also explains the low optical band gap value.

The structure of the as-obtained oligo-pyrazole was also confirmed by the FT-IR, ^1^H NMR, and ^13^C NMR spectra. The FT-IR bands at 3385 cm^−1^ correspond to the -NH amide groups. The bands corresponding to the amide (C=O) groups appear in the region of 1652–1731 cm^−1^. The band observed at 3066 cm^−1^ was attributed to the aromatic C-H stretching vibrations. The strong bands at 1595 and 1579 cm^−1^ correspond to the C-C stretching of the phenyl rings. In the case of the as-synthesized oligo-pyrazole **4**, the correct structure was established by ^1^H NMR and ^13^C NMR spectroscopy in which the characteristic peak for the C=NH proton in was observed at δ = 9.94 ppm and the NH protons were observed as singlet peaks at *δ* = 6.0 and 5.1 ppm, and as multiplets in the region of *δ* = 6.9–8.0 due to the aromatic protons in the as-synthesized oligo-pyrazole **4**.

### The optical properties of the thin films

**Fig. 2:**
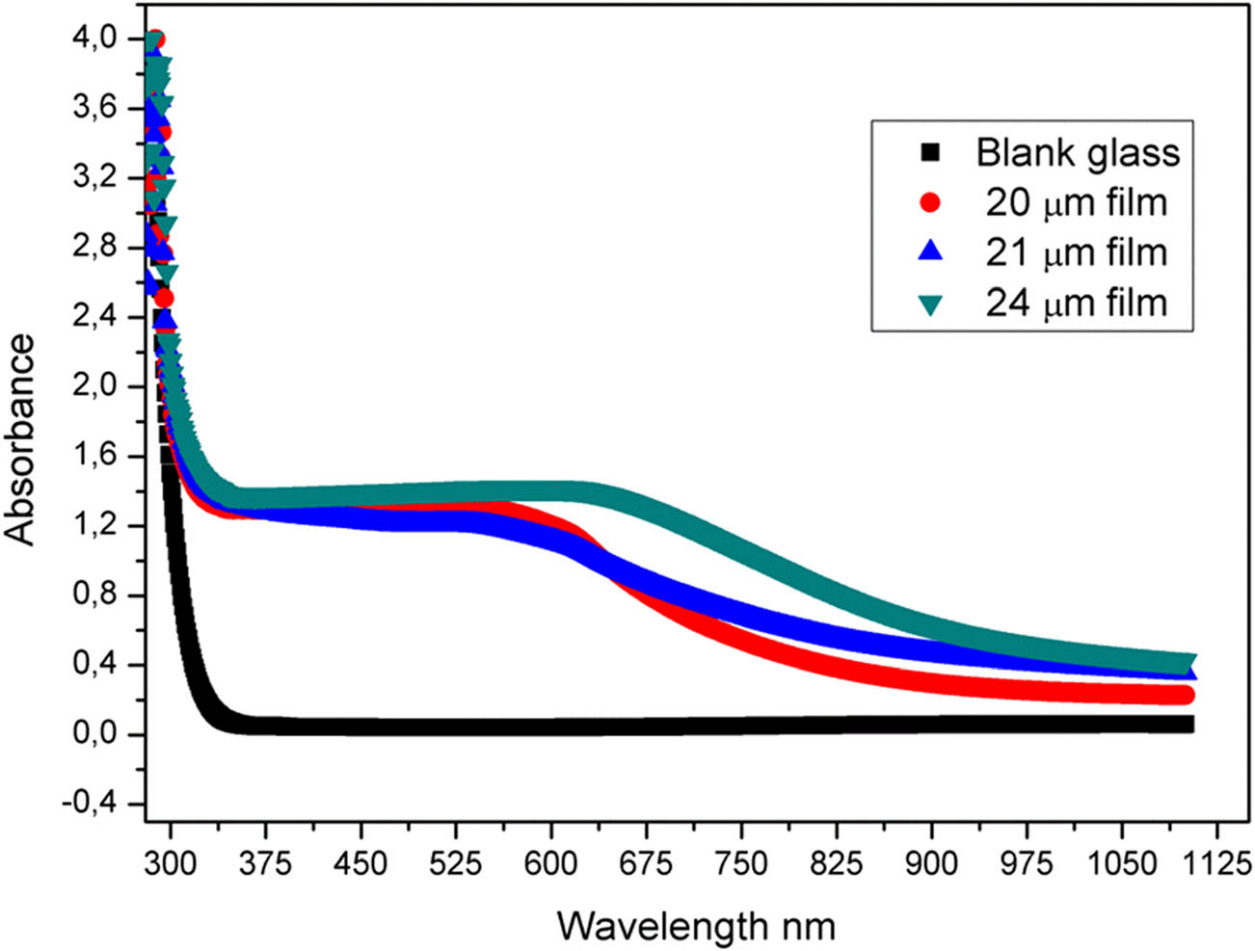
The graph of absorbance vs wavelength obtained for the oligo-pyrazole-coated and uncoated glass samples

The absorbance curves recorded for the oligo-pyrazole coated glass samples and uncoated blank glass were recorded and shown in Fig. 2. The absorbance value of the oligo-pyrazole coated glass samples was increased when compared to the blank glass sample [23]. This effect was easily observed at all the wavelengths studied. In particular, the absorption between 550 and 900 nm increased sharply. An increase in the absorbance value was also observed for the thin films with different thickness levels.

**Fig. 3:**
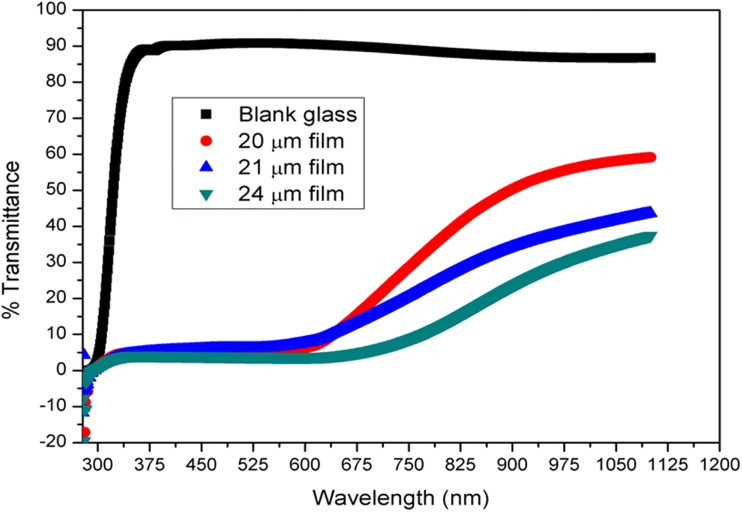
The graph of transmittance vs wavelength obtained for the oligo-pyrazole-coated and uncoated glass samples

The increase in absorbance could be greater if the oligo-pyrazole film was thick. The highest absorbance value was observed for the 24 μm film, while the lowest absorbance value was observed for the 20 μm film. The transmittance graph obtained for the 20, 21, and 24 μm films is shown in Fig. 3.

**Fig. 4:**
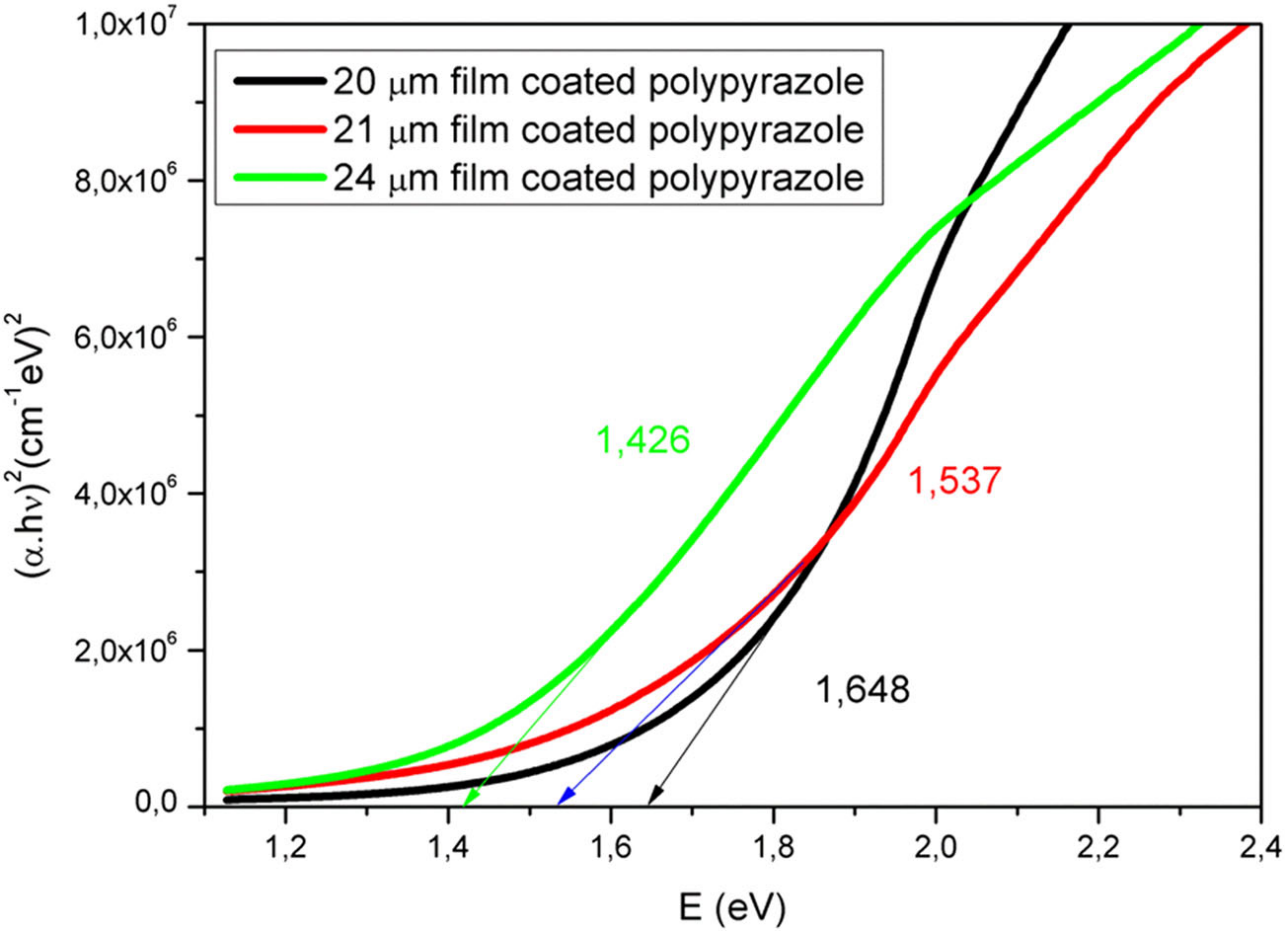
The graph of (αhν)^2^ vs photon energy obtained for oligo-pyrazole

After coating the glass substrate with oligo-pyrazole, the transmittance was significantly reduced. The transmittance value of the blank glass sample was in the range of 90–100% (Fig. 3). Upon coating the glass substrate with oligo-pyrazole, the transmittance value was at most 55%. The transmittance value for the oligo-pyrazole-coated films remained constant in the range of 300–550 nm, whereas it increased in the range of 550–1100 nm. The average transmittance values of the 20, 21, and 24 μm oligo-pyrazole films were 26, 19, and 13% between 294 and 1100 nm, respectively. The average transmittance values of the same films were calculated 12, 10, and 5% in the visible range (between 380 and 780 nm), also respectively. As a result, the transmittance value decreased after increasing the film thickness [24]. Also, we can say that the transparent quality of the oligo-pyrazole films is very low in the visible region due to the remarkably low light transmission [25]. One of the most important parameters for the optical properties of a film coating is the *Eg* value. The photon energy vs (α.hν)^2^ plot was used to obtain the direct band gap of oligo-pyrazole using the Tauc equation (Fig. 4) [26].

**Fig. 5:**
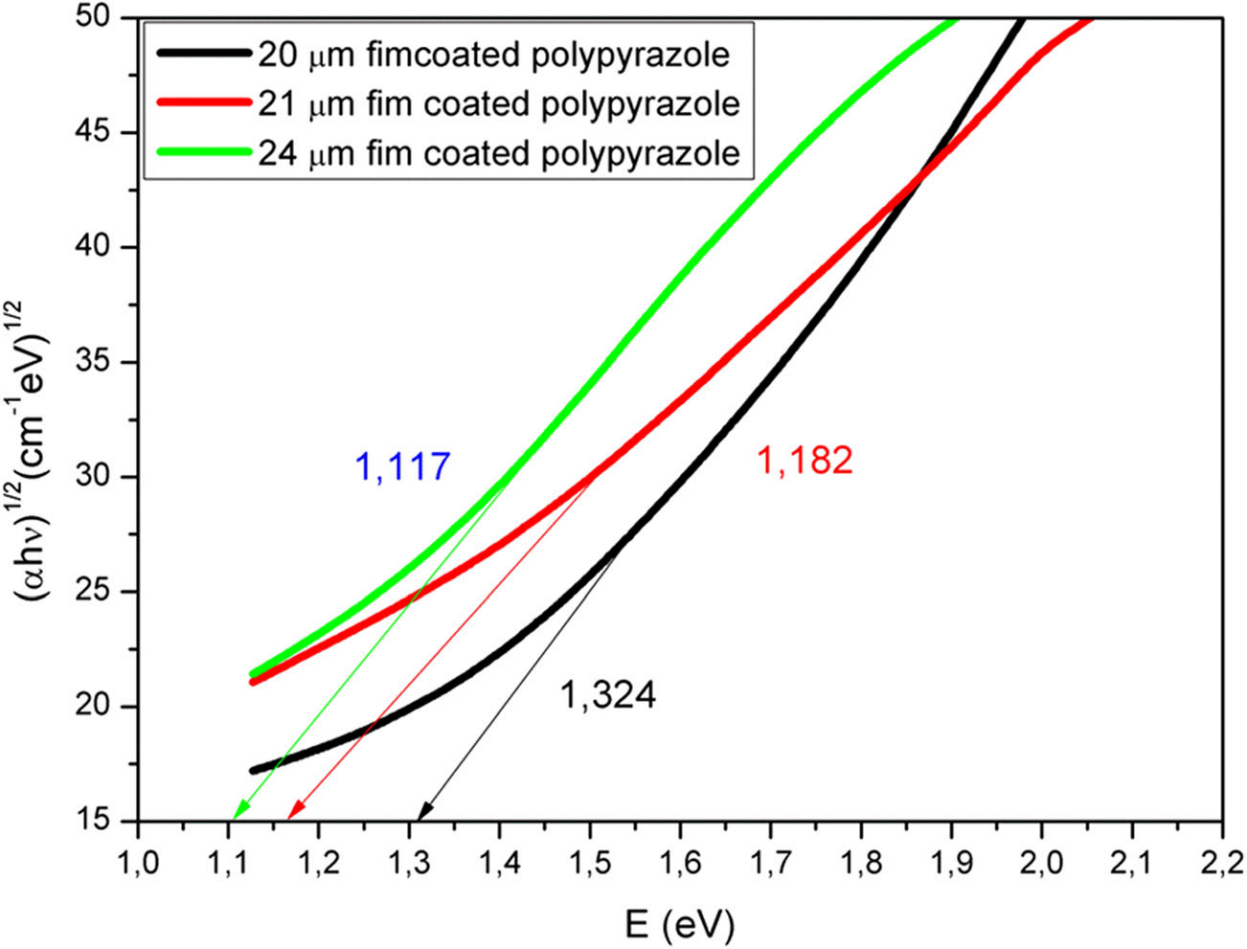
The graph of (αhν)^1/2^ vs photon energy obtained for the thin films of oligo-pyrazole

As shown in Fig. 4, the *E*_*g*_ values observed for the films with thickness values of 20, 21, and 24 μm were 1.646, 1.537, and 1.428 eV, respectively. The *E*_*g*_ value decreased upon increasing the thickness of the oligo-pyrazole film and it was possible to further reduce the *E*_*g*_ value by increasing the film thickness further. In addition, the *E*_*g*_ value of the newly synthesized oligo-pyrazole was very low. When the coated oligo-pyrazole film is set to the desired thickness, it acts as the semiconducting material [27]. Obviously, the desired *E*_*g*_ value can be obtained for a particular optical application by adjusting the film thickness. The photon energy vs (α.hν)^2^ plot was used to determine the indirect *E*_*g*_ values for the thin films of oligo-pyrazole (Fig. 5).

The indirect band gap values observed for the films with thickness values of 20, 21, and 24 μm were found to be 1.324, 1.182, and 1.117 eV, respectively. The *E*_*g*_ value decreased after increasing the film thickness (Fig. 5). The direct transition was sharper than the indirect transition (Figs. 4 and 5) and the direct transition was more dominant than the indirect transition. The direct *E*_*g*_ and indirect *E*_*g*_ values observed for the oligo-pyrazole films with different thickness levels are shown in Table 1.

**Table 1:** The *E*_*g*_ values obtained for the oligo-pyrazole films with thickness values of 20, 21, and 24 μm

No	Thickness (μm)	Direct *E*_*g*_ values (eV)	Indirect *E*_*g*_ values (eV)	Forbidden indirect *Eg* values (eV)
1	20	1648	1324	1466
2	21	1537	1182	1339
3	24	1426	1117	1145

**Fig. 6:**
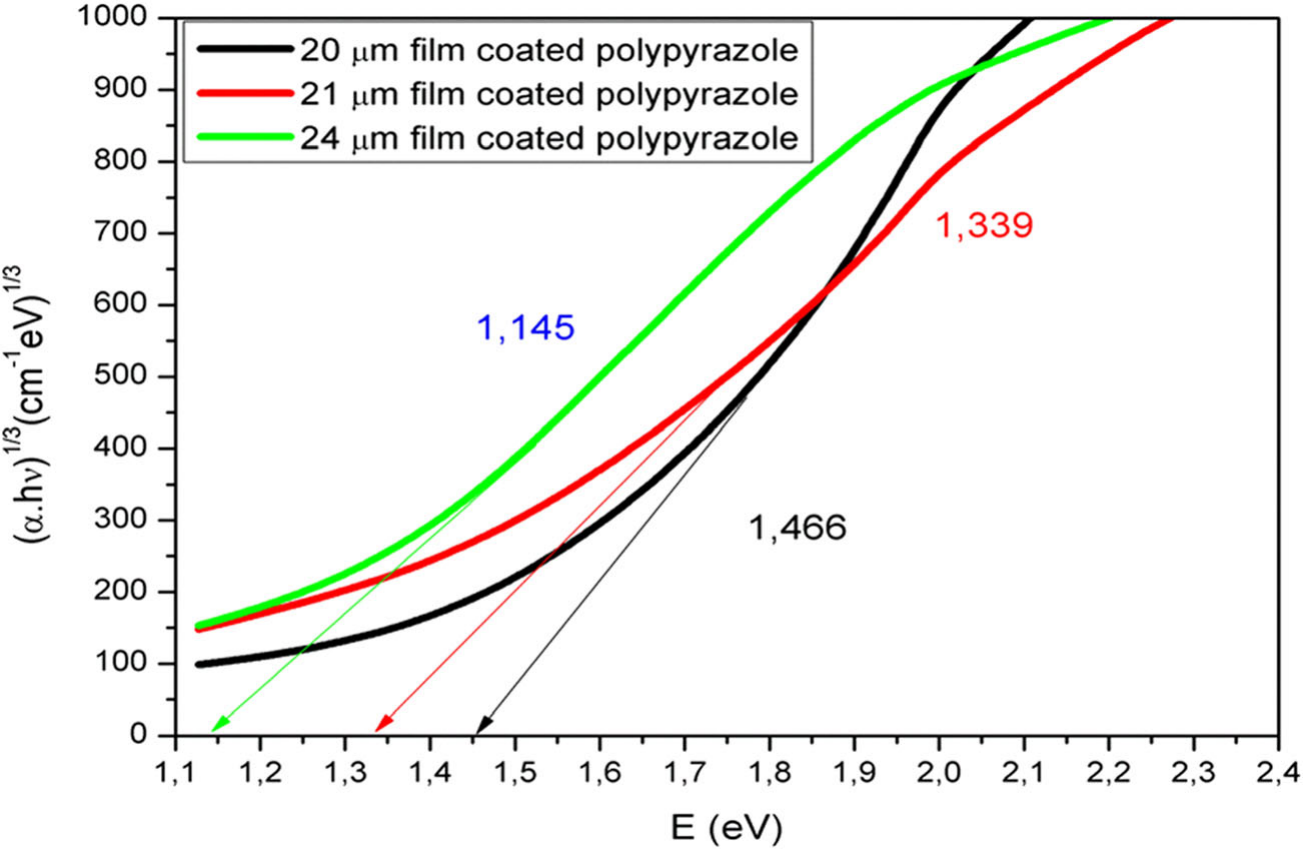
The graph of (αhν)^1/3^ vs photon energy obtained for the thin films of oligo-pyrazole

The graph of *E* (eV) vs (αhν)^1/3^ was plotted for the oligo-pyrazole films with thickness values of 20, 21, and 24 μm (Fig. 6). The forbidden indirect band gap values observed for the 20, 21, and 24 μm films were 1.466, 1.339, and 1.145 eV, respectively. The forbidden indirect band gap value decreased after increasing the film thickness.

The optical band gap is an important parameter for semiconductors. It particularly has a shifting effect in the design of semiconductor materials [28]. For this reason, it is very important that these materials may be modeled and have the desired optical energy gap value. Also, organic photovoltaic devices are among the most popular research areas, because the organic molecules used in these devices have certain properties that are superior to photovoltaic applications. These properties include being low cost, flexible, light weight, and suitable for many new designs [29, 30]. The oligo-pyrazole compound has a very low band gap value, although the repeating unit numbers were very small. This low value (1.42–1.64 eV) is desirable for photovoltaic applications [31]. The minimum band gap value of organic materials that were studied in photovoltaic applications was 1.6–2.2 eV [32, 33]. It appears that the oligo-pyrazole compound has a low *Eg* value as the polymers used in photovoltaic applications. Thus, the synthesized oligo-pyrazole molecule has a potential to be used in photovoltaic applications. The oligo-pyrazole molecule increases the thermal stability by lowering the internal energy because it shows high conjugation [34].

### The surface morphology of thin film

**Fig. 7:**
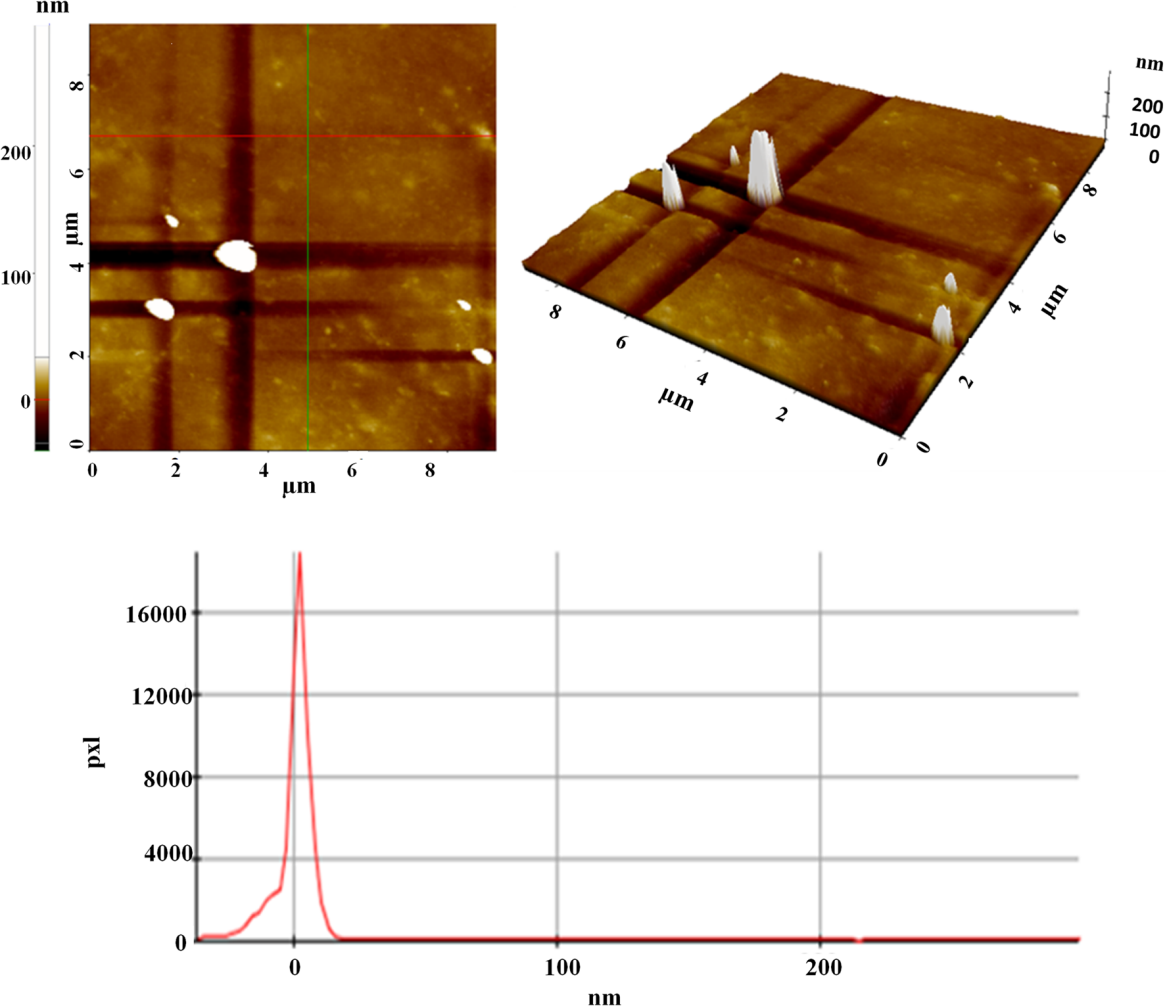
The 2D and 3D AFM images of the as-synthesized oligo-pyrazole thin film coated on a glass substrate

In this study, AFM was used to investigate the surface properties of the oligo-pyrazole thin films. Two- and three-dimensional images (9 μm × 9 μm) of the surface morphologies of the thin films deposited on the glass substrate were recorded at a scanning speed of 0.7 Hz using the AFM device in non-contact mode [35]. The surface features, such as the surface roughness, skewness, kurtosis, and height, were determined using the software on the AFM instrument. Figure 7 shows the two-dimensional and three-dimensional AFM image of the oligo-pyrazole thin film 21 μm coated on a glass substrate [36, 37]. In the AFM images, a few black regions and several yellow regions appeared over a large area. The surface properties of the films were analyzed using high-resolution AFM imaging and image processing evaluation software. The AFM surface analysis results obtained for the thin film coated on the glass substrate showed the average surface roughness, which gives the deviation in height, was 3.34 nm; the average square root roughness, which represents the standard deviation of the surface height, was 5.65 nm and the skewness, which represents the symmetry, was − 0.26 nm. The Kurtosis value, which is a measure of pressure, was 8.84 nm. The total roughness, which is the sum of the maximum height and the maximum depth for the entire measurement length, was 43.80 nm for the red line in the 2D-AFM image, average surface roughness (3.90 nm), average square root roughness (5.74 nm), and skewness (1.18). The Kurtosis value was 5.43. The total roughness was 33.79 nm for the green line in the 2D-AFM graph, as shown in Fig. 6. The skewness value in the produced film was negative for the red line, which showed that the pits on the film surface were more dominant than the peaks.

The height distribution graph was obtained using the AFM tool (Fig. 7). The height distribution plot is associated with the homogeneous grain distribution observed on the samples’ surface. The thin film coated on the glass substrate exhibits a perfect Gauss curve [38]. To summarize, three thin films were obtained with different thickness levels and their optical properties were examined. Desired optical properties can be achieved by adjusting the oligo-pyrazole film thickness.

## Conclusions

This study synthesized a novel conjugated oligomer that was comprised of pyrazole skeleton. Thin films made from this oligomer with different thickness (20, 21, and 24 μm) were prepared and their optical properties were investigated. The band gap values of the oligo-pyrazole films were very low (1.426, 1.537, and 1.648 eV, respectively) and were suitable for optical applications. Furthermore, the band gap value was observed to decrease after increasing the thickness of the thin film, which showed that their optical applications could be controlled by adjusting the oligo-pyrazole film thickness in order to adjust the desired band gap. AFM was used to examine the surface morphology and properties of the organic films, and the two-dimensional and three-dimensional AFM images were used to see the gaps on the surface of the samples. The surfaces of the oligo-pyrazole films have extremely low roughness values. The as-prepared films exhibit good surface morphologies that are suitable for optoelectronic applications.

## Electronic supplementary material

ESM 1 (PDF 711 kb)
