# Supplementary material for: A novel oligo-pyrazole-based thin film: synthesis, characterization, optical and morphological properties
Source: Colloid Polym Sci. 2018 Jun 5;296(7):1249–57. doi: 10.1007/s00396-018-4342-7 (PMC6006238; doi:10.1007/s00396-018-4342-7)

Electronic Supplementary Information

For

A novel oligo-pyrazole based thin film: Synthesis, characterization, optical and morphological properties

Adnan Cetin[1,*], Adem Korkmaz[2], İshak Bildirici[3]
[1]Muş Alparslan University, Faculty of Education, Department of Sciences, Muş, Turkey
[2]Muş Alparslan University, Technical College Vocational School, Muş, Turkey
[3]Yüzüncü Yıl University, Faculty of Pharmacy Department of Pharmaceutical Chemistry, Van, Turkey

e-mail: adnankimya@gmail.com

CONTENTS

FigureS1. ^{1}H NMR Spectra of 1-(3,4-dimethylphenyl)-5-phenyl-1*H*-pyrazole-3,4-dicarboxylic acid (2) 2

FigureS2. ^{13}C NMR Spectra of 1-(3,4-dimethylphenyl)-5-phenyl-1*H*-pyrazole-3,4-dicarboxylic acid (2) 2

FigureS3. ^{1}H NMR of 1-(3,4-dimethylphenyl)-5-phenyl-1*H*-pyrazole-3,4-dicarbonyl dicholoride (3) 3

FigureS4. ^{13}C NMR Spectra of 1-(3,4-dimethylphenyl)-5-phenyl-1*H*-pyrazole-3,4-dicarbonyl dicholoride (3) 3

FigureS5. FTIR Spectra of Poly(p-phenylene-1-(3,4-dimethylphenyl)-5-phenyl-1*H*-pyrazole-3,4-dicarboxyamide (4) 4

FigureS6. GPC Spectra of Poly(p-phenylene-1-(3,4-dimethylphenyl)-5-phenyl-1*H*-pyrazole-3,4-dicarboxyamide (4) 4

FigureS7. ^{1}H NMR Spectra of Poly(p-phenylene-1-(3,4-dimethylphenyl)-5-phenyl-1*H*-pyrazole-3,4-dicarboxyamide (4) 5

FigureS8. ^{13}C NMR Spectra of Poly(p-phenylene-1-(3,4-dimethylphenyl)-5-phenyl-1*H*-pyrazole-3,4-dicarboxyamide (4) 5

FigureS1. ^{1}H NMR Spectra of 1-(3,4-dimethylphenyl)-5-phenyl-1*H*-pyrazole-3,4-dicarboxylic acid (2)

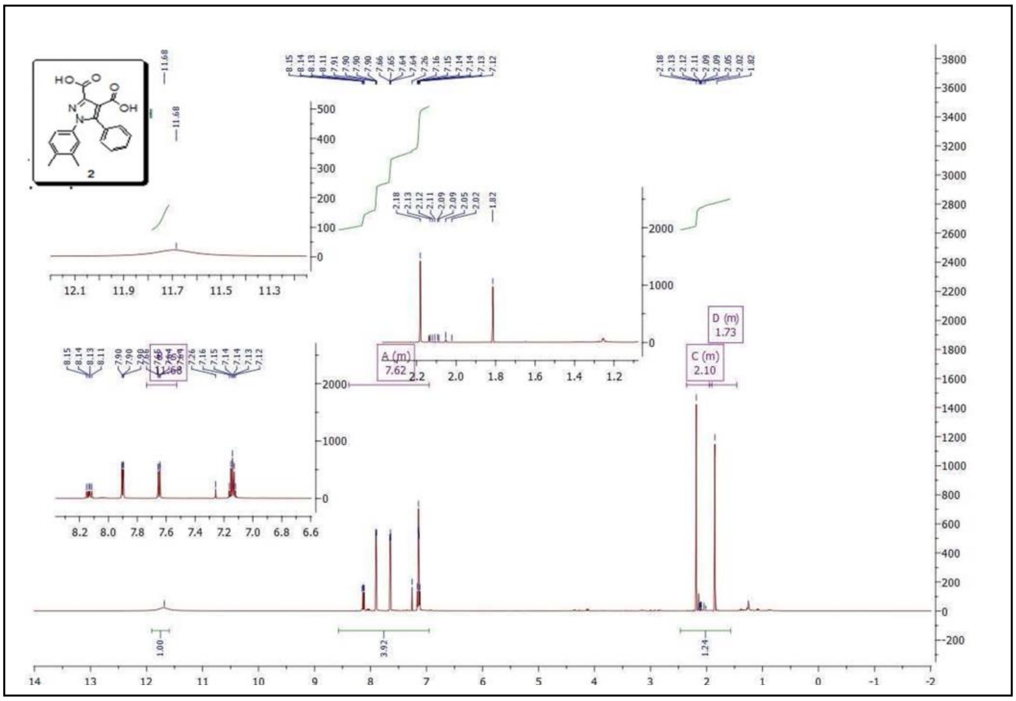

FigureS2. ^{13}C NMR Spectra of 1-(3,4-dimethylphenyl)-5-phenyl-1*H*-pyrazole-3,4-dicarboxylic acid (2)

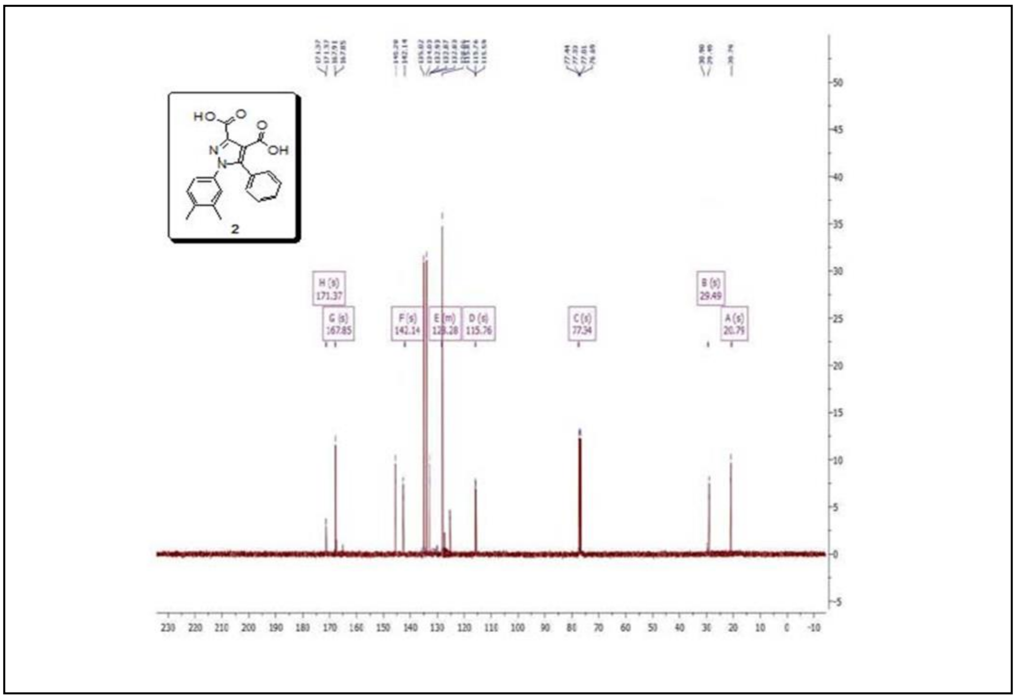

FigureS3. ^{1}H NMR of 1-(3,4-dimethylphenyl)-5-phenyl-1*H*-pyrazole-3,4-dicarbonyl dicholoride (3)

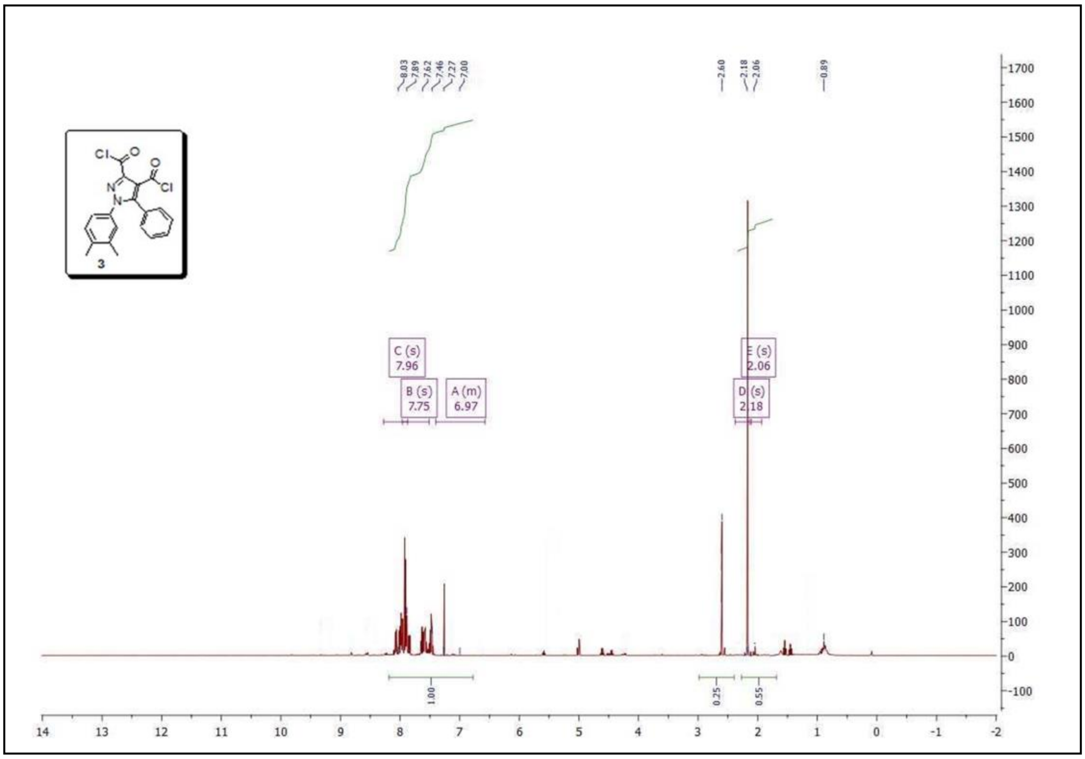

FigureS4. ^{13}C NMR Spectra of 1-(3,4-dimethylphenyl)-5-phenyl-1*H*-pyrazole-3,4-dicarbonyl dicholoride (3)

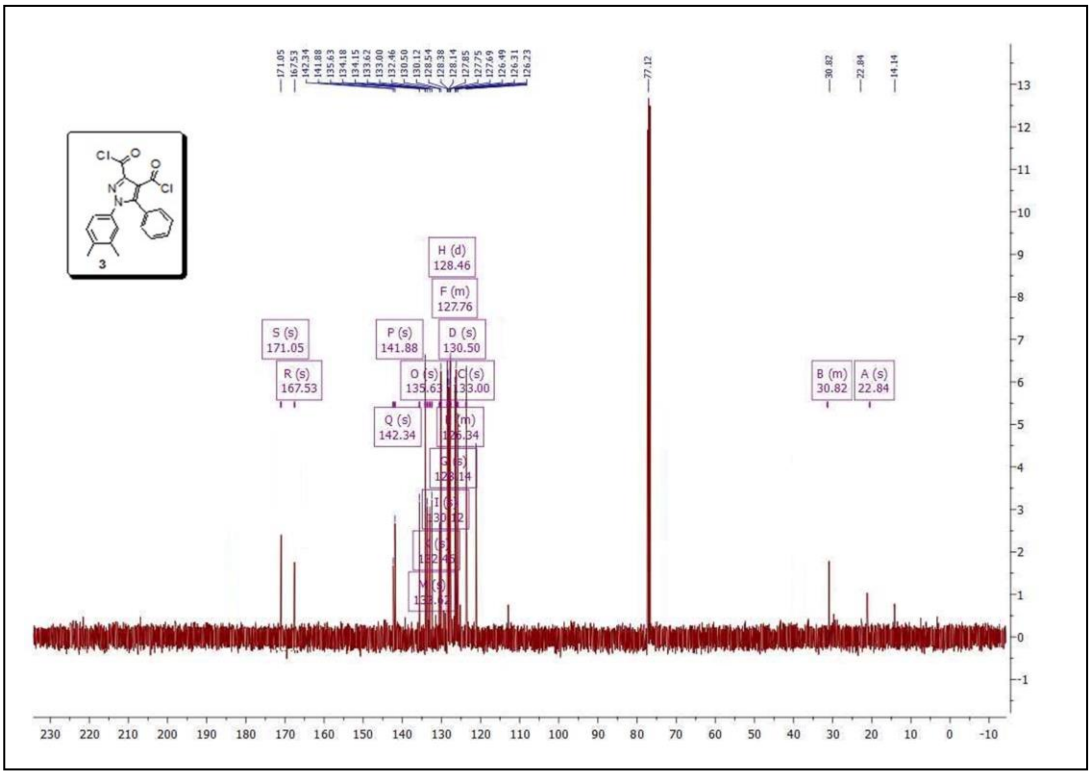

FigureS5. FTIR Spectra of Poly(p-phenylene-1-(3,4-dimethylphenyl)-5-phenyl-1*H*-pyrazole-3,4-dicarboxyamide (4)

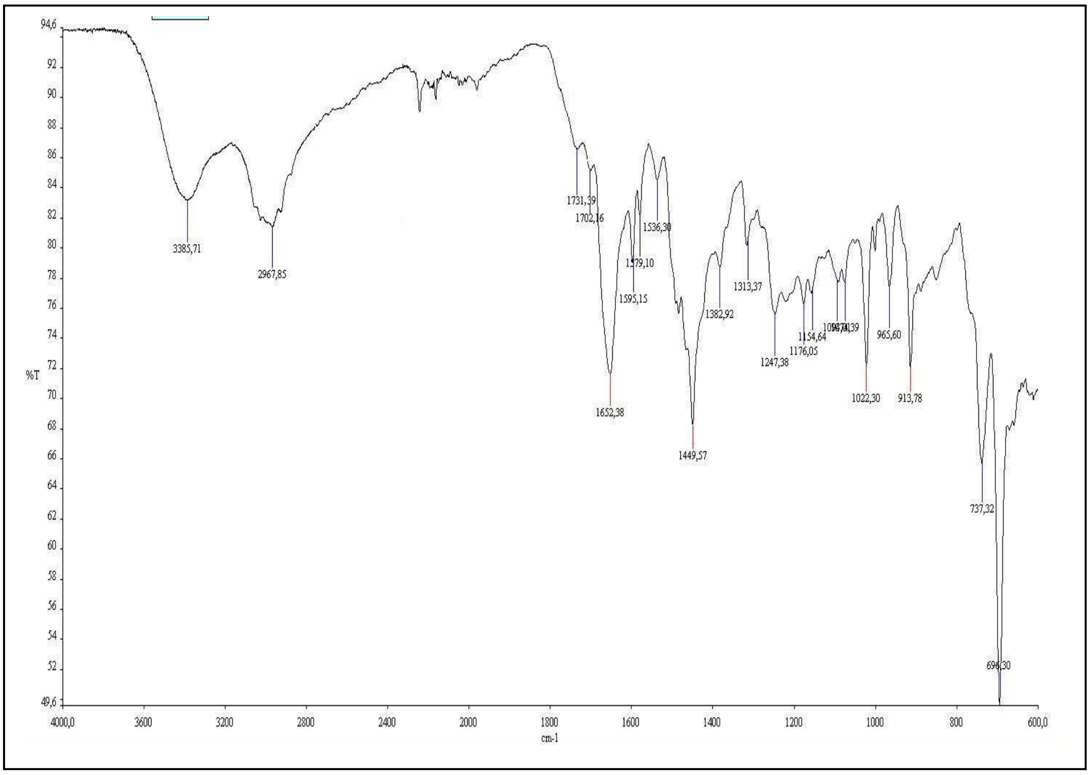

FigureS6. GPC Spectra of Poly(p-phenylene-1-(3,4-dimethylphenyl)-5-phenyl-1*H*-pyrazole-3,4-dicarboxyamide (4)

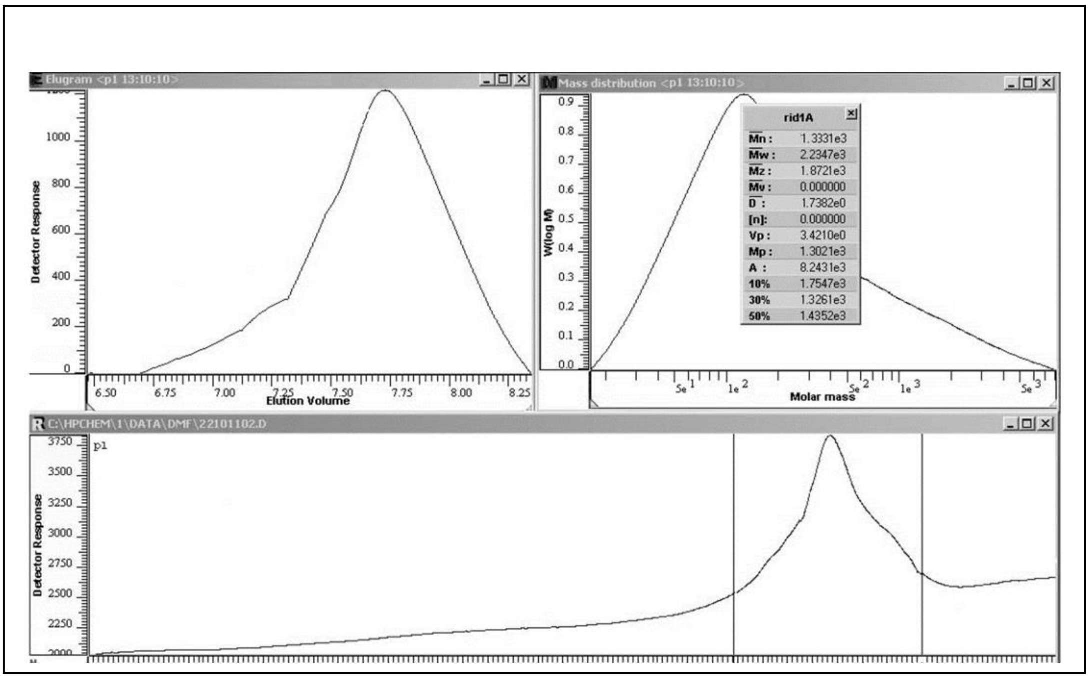

FigureS7. ^{1}H NMR Spectra of Poly(p-phenylene-1-(3,4-dimethylphenyl)-5-phenyl-1*H*-pyrazole-3,4-dicarboxyamide (4)

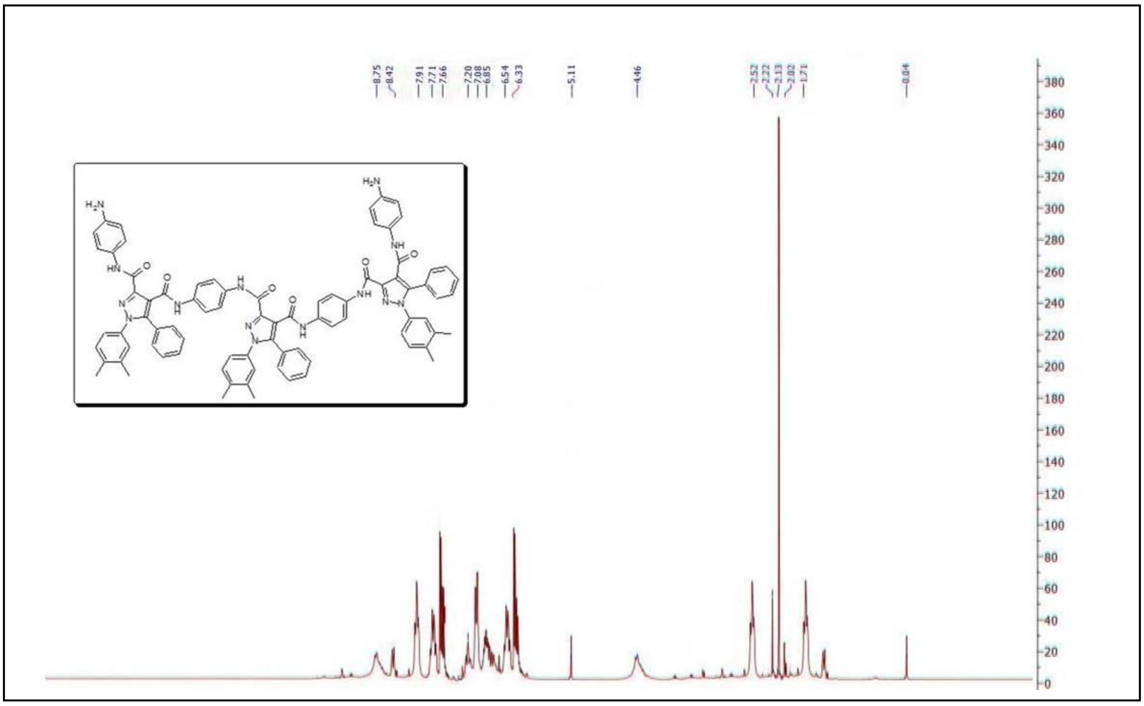

FigureS8. ^{13}C NMR Spectra of Poly(p-phenylene-1-(3,4-dimethylphenyl)-5-phenyl-1*H*-pyrazole-3,4-dicarboxyamide (4)

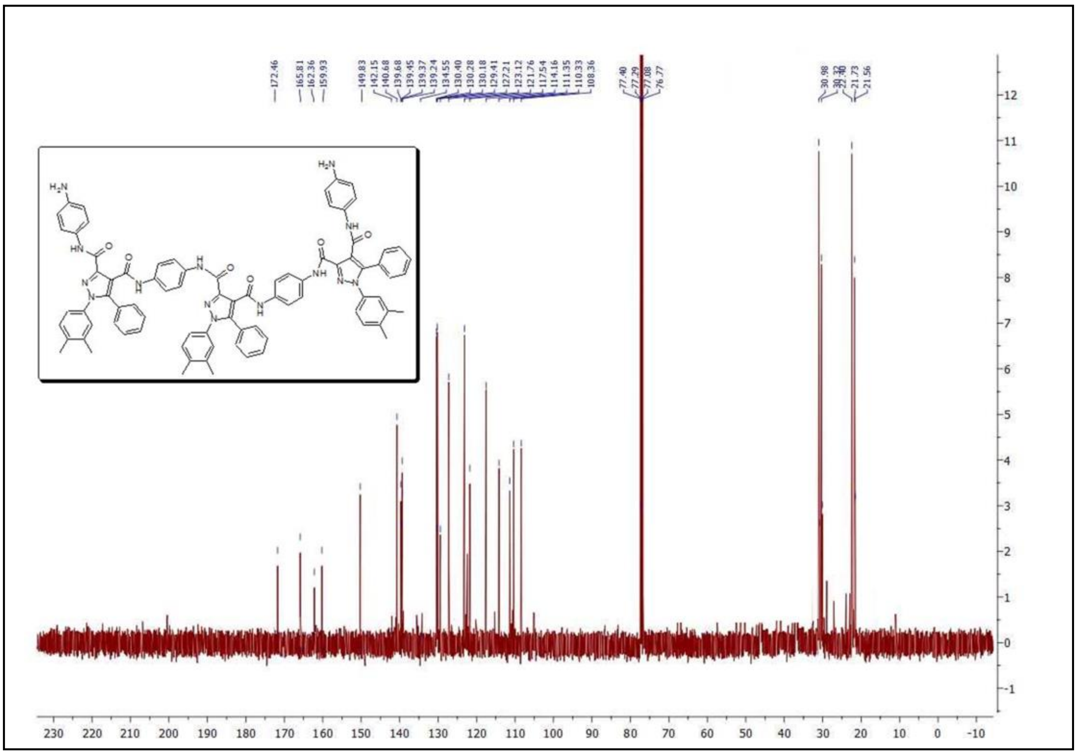

Supplement: Supplementary file 1 — (PDF 711 kb) [file 396_2018_4342_MOESM1_ESM.pdf]
